# Diet Quality and Risk of Bladder Cancer in the Multiethnic Cohort Study

**DOI:** 10.3390/nu16121965

**Published:** 2024-06-20

**Authors:** Minji Kang, Lynne R. Wilkens, Michael D. Wirth, Nitin Shivappa, James R. Hébert, Christopher A. Haiman, Loïc Le Marchand, Song-Yi Park

**Affiliations:** 1 Department of Food and Nutrition, Duksung Women’s University, Seoul 01369, Republic of Korea; 2 Population Sciences in the Pacific Program, University of Hawaii Cancer Center, Honolulu, HI 96813, USA; lynne@cc.hawaii.edu (L.R.W.); loic@cc.hawaii.edu (L.L.M.); spark@cc.hawaii.edu (S.-Y.P.); 3 College of Nursing, University of South Carolina, Columbia, SC 29208, USA; wirthm@mailbox.sc.edu; 4 Department of Epidemiology and Biostatistics, Cancer Prevention and Control Program, Arnold School of Public Health, University of South Carolina, Columbia, SC 29208, USA; shivappa@mailbox.sc.edu (N.S.); jhebert@mailbox.sc.edu (J.R.H.); 5 Department of Nutrition, Connecting Health Innovations LLC, Columbia, SC 29201, USA; 6 Department of Population and Public Health Sciences, Keck School of Medicine and Norris Comprehensive Cancer Center, University of Southern California, Los Angeles, CA 90033, USA; christopher.haiman@med.usc.edu

**Keywords:** bladder cancer, diet quality, multiethnic population, cohort study

## Abstract

This study analyzed the overall quality of the diet using predefined indices, including the Healthy Eating Index-2015 (HEI-2015), the Alternative Healthy Eating Index-2010 (AHEI-2010), the alternate Mediterranean Diet (aMED) score, the Dietary Approaches to Stop Hypertension (DASH) score, and the Dietary Inflammatory Index (DII^®^), to explore their association with the risk of bladder cancer in the Multiethnic Cohort Study. Data were taken from 186,979 African American, Japanese American, Latino, Native Hawaiian, and non-Hispanic White participants aged 45–75 years, with 1152 incident cases of invasive bladder cancer during a mean follow-up period of 19.2 ± 6.6 years. Cox models were used to calculate hazard ratios (HRs) and 95% confidence intervals (CIs) with comprehensive adjustment for smoking. Comparing the highest vs. lowest diet quality score quintile, HRs (95% CIs) in men was 1.08 (0.86–1.36) for HEI-2015, 1.05 (0.84–1.30) for AHEI-2010, 1.01 (0.80–1.27) for aMED, 1.13 (0.90–1.41) for DASH, and 0.96 (0.76–1.21) for DII^®^, whereas the corresponding HRs for women were 0.75 (0.53–1.07), 0.64 (0.45–0.92), 0.60 (0.40–0.88), 0.66 (0.46–0.95), and 0.63 (0.43–0.90) with all *p* values for trend <0.05. The inverse association found in women did not vary by smoking status or race and ethnicity. Our findings suggest that adopting high-quality diets may reduce the risk of invasive bladder cancer among women in a multiethnic population.

## 1. Introduction

Diet has been explored as a preventive strategy for bladder cancer, with strong evidence only found for arsenic in drinking water [1,2,3,4,5,6,7]. Limited evidence suggests that a higher consumption of vegetables, fruits, and tea may decrease the risk of bladder cancer [8]. There is insufficient evidence to draw conclusions regarding the effects of several diet or nutrition factors, including, grains, meat, poultry, fish, milk, alcohol, vitamin A, vitamin C, calcium, and folate, in relation to bladder cancer risk [8]. In approaches that look at overall dietary patterns rather than individual dietary components, an association has been found between high-quality diets and a lower risk of bladder cancer [9]. However, further epidemiological research from more diverse populations is required to confirm the association.

Previously, in the Multiethnic Cohort (MEC), we found that higher intakes of fruits and vegetables, as well as several vitamins including folate, were related to a decreased risk of bladder cancer among women [10]. In this study, with longer follow-up and more cases, we aimed to examine whether high-quality diets, as evaluated by commonly used indices, were associated with bladder cancer risk and whether the associations varied depending on sex, race, and ethnicity, as well as smoking status, a known strong lifestyle risk factor.

## 2. Materials and Methods

### 2.1. Study Population

The MEC is a population-based cohort designed to investigate lifestyle, including diet, and genetic factors associated with cancer and other chronic diseases [11]. Between 1993 and 1996, over 215,000 adults aged 45–75 years who were residents of Hawaii or California completed a self-administered questionnaire. Participants mainly belonged to one of the following five race and ethnic groups, including African American, Japanese American, Latino, Native Hawaiian, or non-Hispanic White. The institutional review boards of the University of Hawaii (CHS9575) and the University of Southern California (HS-17-00714) approved the study protocol. For the present analysis, we excluded individuals who did not identify with any of the five racial and ethnic groups (n = 12,206), those with a history of bladder cancer reported in the baseline questionnaire or tumor registries (n = 525), or those with implausible dietary data regarding energy and macronutrients (n = 8279). Additionally, individuals with incomplete information on cigarette smoking (n = 7638) were excluded. After the exclusions, the final analysis included data from 186,979 participants.

### 2.2. Diet Quality Indices

Participants’ habitual dietary intakes over the previous year were assessed through a quantitative food frequency questionnaire (QFFQ) [11]. The QFFQ included more than 180 food items and was validated through a calibration study, which showed satisfactory correlations (0.57–0.74) for nutrient densities compared to three 24 h dietary recalls [12]. Within the framework of the Dietary Patterns Methods Project, four pre-defined diet quality indices were computed: (1) the Healthy Eating Index-2015 (HEI-2015), (2) the Alternative Healthy Eating Index-2010 (AHEI-2010), (3) the alternate Mediterranean diet (aMED), and (4) Dietary Approaches to Stop Hypertension (DASH) scores [13,14,15]. HEI-2015, a comprehensive scoring system with a potential score range of 0 to 100 points, encompasses 13 components, evaluating adherence to the Dietary Guidelines for Americans 2015–2020 [16]. These components, comprising nine adequacy components and four moderation components, include “total fruits”, “whole fruits”, “total vegetables”, “greens and beans”, “whole grains”, “dairy”, “total protein foods”, “seafood and plant proteins”, “fatty acids”, “refined grains”, “sodium”, “added sugars”, and “saturated fats”. Similarly, AHEI-2010, with a score range of 0 to 110 points, is founded on extensive clinical and epidemiological studies identifying foods and nutrients predictive of chronic disease risk [17]. Its 11 components include “vegetables”, “fruit”, “whole grains”, “sugar-sweetened beverages and fruit juice”, “nuts and legumes”, “red or processed meat”, “trans-fat”, “long-chain omega-3 fats such as EPA, DHA”, “PUFA”, “sodium”, and “alcohol”. The aMED, a scoring system ranging from 0 to 9 points, adapted from the Mediterranean diet score [18], is associated with reduced chronic disease risk [19]. It consists of nine components, including “vegetables”, “legumes”, “fruit”, “nuts”, “whole grains”, “red and processed meats”, “fish”, “ratio of monounsaturated to saturated fat”, and “ethanol”. The DASH score, ranging from 8 to 40 points with eight components, focuses on food and nutrients for managing hypertension [20]. Its eight components encompass “fruits”, “vegetables”, “nuts and legumes”, “whole grains”, “low-fat dairy”, “sodium”, “red and processed meats”, and “sweetened beverages”. Additionally, the Dietary Inflammatory Index (DII^®^) was calculated to determine the overall inflammatory potential of a participant’s diet [21]. Higher scores on the HEI-2015, AHEI-2010, aMED, and DASH indicate better diet quality, while a higher DII^®^ score signifies a more pro-inflammatory diet, reflecting poorer dietary quality. The QFFQ was repeated at a 10-year follow-up survey (2003–2008) [22].

### 2.3. Case Ascertainment

Bladder cancer cases were ascertained by linking the cohort to the tumor registries from the National Cancer Institute Surveillance, Epidemiology, and End Results (SEER) Program for the states of Hawaii and California. Deaths were detected by cross-referencing with death files in both states and the National Death Index. Case and death ascertainment was completed by 31 December 2017. Invasive bladder cancer was explicitly identified using the International Classification of Diseases for Oncology, 3rd edition (ICD-O-3) codes C67.0–C67.9, with exceptions for histology codes 9050–9055, 9140, or 9590–9992, and restricting to invasive malignancies.

### 2.4. Statistical Analysis

Selected baseline characteristics and diet quality scores were presented for men and women with bladder cancer diagnosed during the follow-up period vs. all male and female cohort members. Cox proportional hazard models, utilizing age as the time metric, were employed to calculate hazard ratios (HRs) and 95% confidence intervals (95% CIs) to examine the association between diet quality and the risk of bladder cancer, with separate analyses conducted for men and women. Dietary scores were divided into quintiles based on their distribution in men and women combined. The lowest quintile of the scores, representing the poorest diet quality, served as the reference group, except for the DII^®^, where the highest quintile denoting the most pro-inflammatory diet was used. Trend variables of the scores were determined by the sex- and race and ethnicity-specific medians within each quintile. All models were adjusted for age at cohort entry and log-transformed total energy as covariates and race and ethnicity as a stratification variable. Given the significant association between smoking and bladder cancer, alongside its relationship with diet quality, a comprehensive smoking model developed for the MEC lung cancer study [23] was used with further adjustment for family history of bladder cancer (yes/no) and occupational exposure to high-risk industries (yes/no; employment duration of 10 years or more in rubber or tire manufacturing, plastic production or processing, or pesticide production). The smoking model included smoking status, average number of cigarettes smoked per day, squared average number of cigarettes smoked per day, number of years smoked (time-dependent), number of years since quitting (time-dependent), and interactions of race and ethnicity with smoking status, average number of cigarettes, and squared average number of cigarettes. An a priori selection of covariates was based on their association with bladder cancer risk [24]. We assessed the assumption of proportional hazards using the Schoenfeld residual method and found it met those of [25].

We examined the association stratified by smoking status and race and ethnicity based on a continuous variable of dietary score (per SD). We reversed the DII^®^ score for analysis based on per SD to maintain consistency with other scores. Heterogeneity was evaluated using Wald statistics for the cross-product terms of dietary score variables and subgroup membership variables. Additionally, as part of our supplementary analyses, we updated dietary indices as time-dependent variables based on the QFFQ data from the 10-year follow-up, which were available for 45% of participants in the analytical cohort. We also examined the association using a continuous variable for component scores, which included vegetables, fruit, whole grains, sugar-sweetened beverages and fruit juice, nuts and legumes, red or processed meat, trans-fat, long-chain omega-3 fats, PUFAs, sodium, and alcohol with further adjustments for the modified total score for each component (total score—component i). Data were analyzed using SAS 9.4 software (SAS Institutes, Inc., Cary, NC, USA).

## 3. Results

Among the 186,979 participants, 1152 incident cases of invasive bladder cancer were identified during a mean follow-up of 19.2 (SD = 6.6) years. Compared to same-sex cohort members, men and women diagnosed with bladder cancer were older at cohort entry and more likely to be White, to be former or current smokers, to have a family history of bladder cancer, and to drink alcohol (Table 1). Men diagnosed with bladder cancer had similar or slightly higher diet quality, while women with bladder cancer had marginally lower quality diets compared with the entire cohort of either men or women.

After adjustment for age, race, and ethnicity, as well as total energy intake, the HEI-2015 and DII^®^ score (which were reversed for compatibility with other scores) were inversely associated with bladder cancer risk in men. In contrast, all indices were associated with a lower risk of bladder cancer in women (Table 2). After a comprehensive adjustment for cigarette smoking and other covariates, no statistically significant association was found for any of the indices in men. However, the inverse association with bladder cancer was still significant for all indices (all P’s for trend ≤0.03) in women (all P’s for heterogeneity by sex ≤0.04). When the dietary indices were updated as time-dependent variables using data from the 10-year follow-up survey (Supplementary Table S1), a suggestive inverse association among women was found for all indices but reached statistical significance only for DII^®^ (HRs for Q5 vs. Q1 = 0.67; 95% CI: 0.47–0.97; P for trend = 0.02). Additionally, when examining the association between components of diet quality indices and bladder cancer risk, the inverse association was significant only for the fruit component in women (HRs for an increase of 2 points in the fruit component of the AHEI-2010: 0.90; 95% CI: 0.83–0.97) (Supplementary Table S2).

In subgroup analyses (Table 3), no heterogeneity was found by race and ethnicity or smoking status among men, except for current smokers, who showed a suggestive inverse association between aMED and bladder cancer risk (P for heterogeneity = 0.003). Among women, there was no evidence that the association varied by race and ethnicity or smoking status. However, the number of cases was small, especially for Latino and Native Hawaiian groups.

## 4. Discussion

In this large, multiethnic population, all the dietary indices examined were related to a lower risk of bladder cancer in women. These findings indicate that women who consume high-quality diets have a lower risk of bladder cancer. There was no indication of heterogeneity of effect across smoking status or racial and ethnic groups. However, among men, none of the dietary indices was associated with a lower risk of bladder cancer, either overall or in subgroups, after fully adjusting for smoking, family history of bladder cancer, and occupational exposure.

Previous findings from observational studies on dietary patterns and bladder cancer risk have been inconsistent. A meta-analysis of 12 case–control and cohort studies showed an inverse association with bladder cancer risk for the Mediterranean diet (pooled relative risk, RR = 0.92, 95% CI: 0.87, 0.96) but no significant association for the DII^®^ (RR = 1.04, 95% CI: 0.94, 1.13) [9]. However, a more recent meta-analysis reported a significant increase in bladder cancer with DII^®^ (RR = 1.32, 95% CI: 1.01–1.71) [26]. A population-based study in the US found no association of AHEI-2010 with bladder cancer risk in a case–control design [27] and with overall or bladder cancer-specific survival among invasive bladder cancer cases [28].

Despite the inconsistent results of the previous studies, we found that all five indices, HEI-2015, AHEI-2010, aMED, DASH, and DII^®^, were related to a 25–40% lower risk of bladder cancer among women. Similar to our previous study in the MEC on fruits and vegetables [10], which are common components of the indices included in this work, the inverse association between high-quality diets and bladder cancer risk was confined to women. Several potential underlying mechanisms through which a greater consumption of fruits and vegetables might decrease the risk of bladder cancer are via their high content of dietary fiber, antioxidants, and phytochemicals. These compounds have been found to have anti-carcinogenic properties. Consuming a diet rich in dietary fiber may lower the risk of bladder cancer by promoting bowel regularity and transit time, modulating gut microbiota, and removing damaged cells [2,29,30]. Additionally, antioxidants, such as vitamin C, vitamin E, and beta-carotene, may protect against cancer by helping to protect the cellular system from oxidative damage [31]. Phytochemicals such as flavonoids and polyphenols have anti-inflammatory and antiproliferative effects, which could contribute to their protective role against bladder cancer development [31,32].

While the incidence of cases among women (n = 309) was notably small compared to men (n = 843), the risk reduction observed in women was large enough to reach statistical significance. Overall, diet quality was higher in women than men in the MEC [33], but we used the same cutoffs for men and women to define quantiles for direct comparison. In addition, the dietary indices based on sex-specific distributions such as aMED and DASH did not show a significant association for men. When we examined the association in the models with continuous dietary scores (per SD), an inverse association was observed only in women. Men may have had higher exposure to high-risk industries than women. Although we adjusted for this, the baseline questionnaire might not have fully captured occupation-related risk factors. Our findings suggest that high-quality diets may not be beneficial for the prevention of bladder cancer in men, who are at overall higher risk compared to women.

The current study has several strengths, including its population-based nature, the multiethnic background of participants, its prospective design, long follow-up, comprehensive data on diet, and ability to control for potential confounders. Despite its strengths, there are limitations to be considered when interpreting the results. Although dietary data were collected using a validated QFFQ, they are subject to measurement error. We were able to update dietary data from the 10-year follow-up survey, but this was only available for 45% of the study population. In addition, statistical power was limited for subgroup analyses, especially for Latino and Native Hawaiian women.

## 5. Conclusions

In conclusion, this study’s findings support that high-quality diets lower the risk of invasive bladder cancer among women in a multiethnic population. No evidence was found to prove that the association varied by race and ethnicity or smoking status within the sexes. However, because the number of cases was limited for some racial and ethnic groups in women, further research is warranted for larger ethnic populations with more cases.

## Figures and Tables

**Table 1:** Baseline characteristics of participants by bladder cancer status in the Multiethnic Cohort Study.

	Men		Women	
	Invasive Bladder Cancer	Entire Cohort	Invasive Bladder Cancer	Entire Cohort
No.	843	84,227	309	102,752
Age at cohort entry, mean (SD)	63.4 (7.8)	60.2 (8.9)	62.7 (8.1)	59.7 (8.8)
Age at diagnosis, mean (SD)	76.5 (8.7)		76.2 (9.0)	
Race and ethnicity, n (%)				
African American	109 (12.9)	11,401 (13.5)	78 (25.2)	20,051 (19.5)
Japanese American	244 (28.9)	25,218 (29.9)	73 (23.6)	28,284 (27.5)
Latino	172 (20.4)	20,422 (24.2)	42 (13.6)	21,878 (21.3)
Native Hawaiian	45 (5.3)	5884 (7.0)	24 (7.8)	7595 (7.4)
non-Hispanic White	273 (32.4)	21,302 (25.3)	92 (29.8)	24,944 (24.3)
Education, n (%)				
≤High school	368 (43.7)	34,599 (41.1)	146 (47.2)	47,061 (45.8)
Vocational school/some college	249 (29.5)	24,578 (29.2)	90 (29.1)	30,403 (29.6)
≥College graduate	222 (26.3)	24,747 (29.4)	70 (22.7)	24,785 (24.1)
missing	4 (0.5)	303 (0.4)	3 (1.0)	503 (0.5)
Body mass index, kg/m^2^, mean (SD)	26.3 (3.9)	26.6 (4.2)	26.2 (5.5)	26.5 (5.8)
Smoking status, n (%)				
Never	171 (20.3)	25,977 (30.8)	120 (38.8)	58,480 (56.9)
Former	487 (57.8)	42,910 (50.9)	111 (35.9)	29,559 (28.8)
Current	185 (21.9)	15,340 (18.2)	78 (25.2)	14,713 (14.3)
Pack years of smoking, n (%) ^1^	24.8 (17.0)	20.7 (16.6)	20.3 (15.2)	15.5 (14.4)
High-risk industry, n (%)	13 (1.5)	1412 (1.7)	4 (1.3)	1086 (1.1)
Family history of bladder cancer, n (%)	10 (1.2)	404 (0.5)	6 (1.9)	779 (0.8)
Alcohol, g/day, mean (SD)	16.1 (31.8)	14.7 (32.6)	6.2 (13.7)	4.3 (14.9)
Total energy, kcal/day, mean (SD)	2377 (1157)	2421 (1123)	1939 (997)	1975 (960)
Dietary quality scores ^2^				
HEI-2015, mean (SD)	66.1 (10.4)	65.5 (10.3)	68.3 (11.1)	69.0 (10.4)
AHEI-2010, mean (SD)	64.8 (9.9)	64.2 (9.8)	64.4 (9.3)	65.1 (9.2)
aMED, mean (SD)	4.2 (1.8)	4.2 (1.8)	3.9 (1.8)	4.1 (1.8)
DASH, mean (SD)	24.5 (4.4)	24.1 (4.4)	23.5 (4.3)	24.1 (4.4)
DII^®^, mean (SD)	−0.92 (1.93)	−0.90 (1.97)	−1.49 (2.04)	−1.77 (1.88)

^1^ Among ever-smokers. ^2^ Diet quality scores: HEI-2015, Healthy Eating Index-2015; AHEI-2010, Alternative Healthy Eating Index-2010; aMED, alternate Mediterranean Diet score; DASH, Dietary Approaches to Stop Hypertension; DII^®^, Dietary Inflammatory Index.

**Table 2:** HRs (95% CI) for bladder cancer risk according to the quintiles of the diet quality indices in the Multiethnic Cohort Study, 1993–2017.

Dietary Quality Scores ^1^	Men			Women			P for Heterogeneity ^4^
	Cases	HR (95% CI) ^2^	HR (95% CI) ^3^	Cases	HR (95% CI) ^2^	HR (95% CI) ^3^	
HEI-2015							
17.9 to 58.2	189	1.00 (ref.)	1.00 (ref.)	58	1.00 (ref.)	1.00 (ref.)	
58.3 to 64.6	196	1.01 (0.83–1.23)	1.10 (0.90–1.35)	60	0.82 (0.57–1.18)	0.90 (0.63–1.29)	
64.7 to 70.2	161	0.85 (0.69–1.05)	0.98 (0.79–1.21)	57	0.63 (0.44–0.92)	0.72 (0.50–1.05)	
70.3 to 76.6	154	0.84 (0.68–1.04)	1.01 (0.81–1.26)	49	0.45 (0.31–0.66)	0.54 (0.36–0.80)	
76.7 to 100	143	0.85 (0.68–1.06)	1.08 (0.86–1.36)	85	0.60 (0.43–0.85)	0.75 (0.53–1.07)	
P for trend		0.045	0.77	309	0.0004	0.03	0.0238
AHEI-2010							
25.1 to 56.6	172	1.00 (ref.)	1.00 (ref.)	71	1.00 (ref.)	1.00 (ref.)	
56.7 to 62.2	162	0.93 (0.75–1.15)	1.00 (0.81–1.25)	54	0.64 (0.45–0.91)	0.69 (0.49–0.99)	
62.3 to 67.1	162	0.92 (0.74–1.14)	1.03 (0.83–1.28)	64	0.69 (0.49–0.97)	0.77 (0.55–1.09)	
67.2 to 72.6	169	0.93 (0.75–1.16)	1.09 (0.88–1.36)	60	0.59 (0.41–0.84)	0.68 (0.47–0.96)	
72.7 to 104.5	178	0.87 (0.70–1.08)	1.05 (0.84–1.30)	60	0.55 (0.39–0.79)	0.64 (0.45–0.92)	
P for trend		0.25	0.51		0.001	0.02	0.01
aMED							
0 to 2	158	1.00 (ref.)	1.00 (ref.)	76	1.00 (ref.)	1.00 (ref.)	
3	160	1.04 (0.84–1.30)	1.11 (0.89–1.38)	60	0.85 (0.60–1.19)	0.89 (0.63–1.25)	
4	146	0.82 (0.66–1.04)	0.91 (0.72–1.14)	55	0.68 (0.48–0.97)	0.74 (0.52–1.06)	
5	167	0.97 (0.77–1.21)	1.12 (0.89–1.40)	58	0.71 (0.50–1.03)	0.81 (0.56–1.16)	
6 to 9	212	0.82 (0.66–1.03)	1.01 (0.80–1.27)	60	0.50 (0.34–0.74)	0.60 (0.40–0.88)	
P for trend		0.07	0.94		0.0005	0.01	0.04
DASH							
8 to 20	154	1.00 (ref.)	1.00 (ref.)	79	1.00 (ref.)	1.00 (ref.)	
21 to 22	109	0.93 (0.73–1.19)	1.00 (0.78–1.28)	50	0.84 (0.58–1.19)	0.91 (0.64–1.30)	
23 to 25	237	1.08 (0.88–1.33)	1.25 (1.01–1.54)	73	0.64 (0.46–0.89)	0.74 (0.53–1.02)	
26 to 27	131	0.90 (0.71–1.14)	1.09 (0.85–1.39)	42	0.55 (0.37–0.80)	0.66 (0.44–0.97)	
28 to 40	212	0.87 (0.70–1.08)	1.13 (0.90–1.41)	65	0.53 (0.37–0.75)	0.66 (0.46–0.95)	
P for trend		0.15	0.28		0.0001	0.02	0.006
DII^®^							
0.46 to 4.98	211	1.00 (ref.)	1.00 (ref.)	57	1.00 (ref.)	1.00 (ref.)	
−0.94 to 0.45	224	1.11 (0.92–1.34)	1.23 (1.02–1.49)	63	0.81 (0.57–1.17)	0.90 (0.63–1.30)	
−2.12 to −0.95	157	0.85 (0.69–1.05)	1.00 (0.81–1.23)	58	0.58 (0.40–0.84)	0.69 (0.47–1.00)	
−3.24 to −2.13	134	0.79 (0.63–0.98)	0.96 (0.77–1.20)	59	0.48 (0.33–0.69)	0.60 (0.41–0.87)	
−6.44 to −3.25	117	0.75 (0.60–0.95)	0.96 (0.76–1.21)	72	0.49 (0.34–0.70)	0.63 (0.43–0.90)	
P for trend		0.0008	0.34		<0.0001	0.002	0.01

^1^ Diet quality scores: HEI-2015, Healthy Eating Index-2015; AHEI-2010, Alternative Healthy Eating Index-2010; aMED, alternate Mediterranean Diet score; DASH, Dietary Approaches to Stop Hypertension; DII^®,^ Dietary Inflammatory Index^®^. ^2^ Adjusted for age at cohort entry, race and ethnicity, and total energy intake. ^3^ Further adjusted for family history of bladder cancer, employment in a high-risk industry, smoking status, average number of cigarettes, squared average number of cigarettes, number of years smoked (time-dependent), number of years since quitting (time-dependent), interactions of race/ethnicity with smoking status, average number of cigarettes, squared average number of cigarettes, and number of years smoked. HEI-2015 and DASH were additionally adjusted for alcohol intake. ^4^ Based on the fully adjusted model.

**Table 3:** HRs (95% CIs) for invasive bladder cancer per SD of diet quality indices overall and subgroups by race and ethnicity and smoking status in the Multiethnic Cohort Study, 1993–2017 ^1,2^.

	Cases	HEI-2015	AHEI-2010	aMED	DASH	DII^® 3^
Men	843	1.01 (0.94–1.08)	1.02 (0.95–1.10)	1.00 (0.93–1.08)	1.03 (0.96–1.11)	0.96 (0.89–1.03)
Race and ethnicity						
African American	109	1.17 (0.96–1.42)	1.18 (0.96–1.44)	1.08 (0.87–1.34)	1.21 (0.98–1.49)	1.16 (0.95–1.42)
Japanese American	244	0.95 (0.83–1.09)	0.97 (0.85–1.10)	1.01 (0.87–1.17)	1.10 (0.96–1.26)	0.94 (0.83–1.08)
Latino	172	0.95 (0.80–1.13)	0.98 (0.82–1.16)	0.85 (0.71–1.02)	0.92 (0.77–1.11)	0.85 (0.72–1.01)
Native Hawaiian	45	1.00 (0.74–1.34)	1.17 (0.87–1.59)	1.20 (0.86–1.69)	0.97 (0.71–1.32)	1.00 (0.75–1.35)
White	273	1.03 (0.91–1.16)	1.04 (0.92–1.17)	1.05 (0.92–1.19)	0.99 (0.87–1.13)	0.94 (0.83–1.07)
P for heterogeneity ^4^		0.46	0.72	0.55	0.70	0.25
Smoking status						
Never	171	1.03 (0.88–1.21)	1.08 (0.92–1.27)	1.06 (0.90–1.26)	1.06 (0.89–1.25)	0.97 (0.83–1.14)
Former	487	1.04 (0.94–1.14)	1.05 (0.96–1.15)	1.04 (0.94–1.15)	1.06 (0.96–1.17)	0.98 (0.89–1.08)
Current	185	0.92 (0.79–1.07)	0.93 (0.80–1.08)	0.86 (0.73–1.02)	0.95 (0.80–1.12)	0.90 (0.77–1.06)
P for heterogeneity ^4^		0.19	0.11	0.003	0.14	0.36
Women	309	0.86 (0.77–0.97)	0.88 (0.79–0.99)	0.84 (0.74–0.96)	0.83 (0.73–0.94)	0.83 (0.74–0.93)
Race and ethnicity						
African American	78	0.77 (0.62–0.97)	0.83 (0.66–1.04)	0.74 (0.57–0.96)	0.77 (0.60–0.98)	0.74 (0.59–0.92)
Japanese American	73	0.79 (0.62–1.00)	0.96 (0.75–1.23)	0.94 (0.71–1.24)	0.93 (0.72–1.19)	0.90 (0.71–1.15)
Latino	42	1.24 (0.89–1.72)	1.17 (0.83–1.64)	1.18 (0.82–1.70)	1.00 (0.71–1.41)	0.90 (0.65–1.25)
Native Hawaiian	24	0.70 (0.47–1.06)	0.77 (0.52–1.16)	0.69 (0.43–1.12)	0.95 (0.62–1.46)	0.76 (0.52–1.11)
White	92	0.94 (0.76–1.16)	0.84 (0.68–1.03)	0.79 (0.63–1.00)	0.73 (0.58–0.91)	0.87 (0.71–1.07)
P for heterogeneity ^4^		0.11	0.49	0.63	0.51	0.81
Smoking status						
Never	120	0.84 (0.69–1.01)	0.90 (0.74–1.09)	0.86 (0.70–1.06)	0.83 (0.68–1.01)	0.82 (0.68–0.99)
Former	111	0.96 (0.79–1.18)	0.89 (0.74–1.08)	0.93 (0.75–1.16)	0.88 (0.72–1.09)	0.93 (0.76–1.13)
Current	78	0.78 (0.62–0.97)	0.85 (0.68–1.06)	0.69 (0.53–0.90)	0.76 (0.60–0.98)	0.73 (0.59–0.90)
P for heterogeneity ^4^		0.44	0.98	0.38	0.65	0.34

^1^ Diet quality scores: HEI-2015, Healthy Eating Index-2015; AHEI-2010, Alternative Healthy Eating Index-2010; aMED, alternate Mediterranean Diet score; DASH, Dietary Approaches to Stop Hypertension; DII^®,^ Dietary Inflammatory Index^®^. ^2^ Adjusted for age at cohort entry, race/ethnicity, family history of bladder cancer, employment in a high-risk industry, and total energy intake in the smoking model, including smoking status, average number of cigarettes, squared average number of cigarettes, number of years smoked (time-dependent), number of years since quitting (time-dependent), interactions of race/ethnicity with smoking status, average number of cigarettes, squared average number of cigarettes, and number of years smoked. HEI-2015 and DASH were additionally adjusted for alcohol intake. ^3^ DII^®^ score was reversed for analysis based on per SD to maintain consistency with other scores. ^4^ Tests for heterogeneity by subgroups were based on the Wald statistics for cross-product terms of continuous variables for diet quality indices and subgroups.

## Data Availability

The data presented in this study are available on request, pending application to and approval by the Multiethnic Cohort Research Committee (https://www.uhcancercenter.org/for-researchers/mec-data-sharing, accessed on 1 April 2024).

## Supplementary Materials

The following supporting information can be downloaded at: https://www.mdpi.com/article/10.3390/nu16121965/s1, Table S1: Diet quality indices as time-dependent variables and bladder cancer risk in the Multiethnic Cohort Study; Table S2: Components of diet quality indices and bladder cancer risk in the Multiethnic Cohort Study.
