# Supplementary material for: Diet Quality and Risk of Bladder Cancer in the Multiethnic Cohort Study
Source: Nutrients. 2024 Jun 20;16(12):1965. doi: 10.3390/nu16121965 (PMC11206544; doi:10.3390/nu16121965)
Supplement: Supplementary file 1 [file nutrients-16-01965-s001.zip › nutrients-3035941-supplementary.pdf]

Supplementary Tables

Supplementary Table S1. Diet quality indices as time-dependent variables and bladder cancer risk in the Multiethnic Cohort Study

	Men (n=84,227)		Women (n=102,752)		
	Cases	HR (95% CI)	Cases	HR (95% CI)	P for heterogeneity
HEI-2015					
17.9 to 58.2	171	1.00 (ref.)	59	1.00 (ref.)	
58.3 to 64.6	178	1.06 (0.86-1.31)	47	0.69 (0.47-1.02)	
64.7 to 70.2	161	1.00 (0.80-1.24)	56	0.69 (0.47-1.00)	
70.3 to 76.6	169	1.10 (0.88-1.37)	52	0.54 (0.37-0.80)	
76.7 to 100	164	1.09 (0.87-1.37)	95	0.74 (0.53-1.04)	
P for trend		0.4168		0.1230	0.0402
AHEI-2010					
25.1 to 56.6	165	1.00 (ref.)	63	1.00 (ref.)	
56.7 to 62.2	145	0.92 (0.74-1.15)	52	0.77 (0.53-1.12)	
62.3 to 67.1	155	0.99 (0.80-1.24)	69	0.96 (0.68-1.35)	
67.2 to 72.6	176	1.10 (0.89-1.37)	56	0.71 (0.49-1.03)	
72.7 to 104.5	202	1.04 (0.84-1.29)	69	0.75 (0.53-1.07)	
P for trend		0.3663		0.1197	0.0445
aMED					
0 to 2	140	1.00 (ref.)	72	1.00 (ref.)	
3	167	1.32 (1.05-1.65)	62	1.01 (0.72-1.42)	
4	165	1.19 (0.95-1.49)	50	0.77 (0.53-1.10)	
5	154	1.19 (0.95-1.50)	64	1.05 (0.75-1.47)	
6 to 9	217	1.18 (0.95-1.46)	61	0.74 (0.52-1.05)	
P for trend		0.4434		0.1451	0.0703
DASH					
8 to 20	139	1.00 (ref.)	73	1.00 (ref.)	
21 to 22	100	0.93 (0.72-1.21)	41	0.78 (0.53-1.15)	
23 to 25	218	1.13 (0.91-1.41)	78	0.83 (0.60-1.15)	
26 to 27	141	1.14 (0.89-1.44)	40	0.66 (0.44-0.97)	
28 to 40	245	1.19 (0.96-1.48)	77	0.78 (0.56-1.09)	
P for trend		0.0538		0.1764	0.0145
DII					
0.46 to 4.98	187	1.00 (ref.)	50	1.00 (ref.)	
-0.94 to 0.45	205	1.15 (0.94-1.41)	57	0.90 (0.61-1.31)	
-2.12 to -0.95	153	0.95 (0.77-1.18)	55	0.70 (0.47-1.03)	
-3.24 to -2.13	141	0.95 (0.76-1.18)	63	0.67 (0.45-0.97)	
-6.44 to -3.25	157	1.05 (0.84-1.31)	84	0.67 (0.47-0.97)	
P for trend		0.7758		0.0157	0.0223

Diet quality indices were updated as time-dependent variables using data from the 10-year follow-up questionnaire for 84,223 participants who had no bladder cancer prior to the 10-year follow-up survey.

Adjusted by Cox regression with age as the time metric for age at cohort entry, race and ethnicity, family history of bladder cancer, employment in a high-risk industry, and total energy intake in the smoking model. For HEI-2015 and DASH, additionally adjusted for alcohol intake.

Supplementary Table S2. Components of diet quality indices and bladder cancer risk in the Multiethnic Cohort Study

	Men (n=84,227)		Women (n=102,752)	
	HR (95% CI)	P-value	HR (95% CI)	P-value
AHEI-2010				
Vegetables	0.97 (0.91-1.03)	0.2595	0.93 (0.84-1.03)	0.1736
Fruits	0.99 (0.95-1.04)	0.6875	0.90 (0.83-0.97)	0.0071
Whole grains	0.99 (0.93-1.06)	0.8376	0.97 (0.87-1.07)	0.4842
Sugar-sweetened beverages and fruit juice*	1.03 (0.99-1.07)	0.1078	0.99 (0.93-1.05)	0.7218
Nuts and legumes	1.01 (0.95-1.07)	0.7922	0.93 (0.85-1.01)	0.0912
trans Fat*	1.09 (0.69-1.73)	0.7091	0.70 (0.37-1.34)	0.2854
Long-chain (n-3) fats (EPA + DHA)	0.96 (0.91-1.02)	0.1602	1.03 (0.94-1.13)	0.5267
PUFA	1.01 (0.95-1.08)	0.6820	0.99 (0.89-1.10)	0.8504
Alcohol*	1.00 (0.96-1.05)	0.8286	1.05 (0.96-1.15)	0.2800
Sodium*	1.07 (0.99-1.17)	0.1013	1.02 (0.88-1.17)	0.8054
Red/processed meat*	1.01 (0.95-1.07)	0.8316	0.99 (0.88-1.11)	0.8654

HR for 2 points increase in component score.
Adjusted for age at cohort entry, race and ethnicity, family history of bladder cancer, employment in a high-risk industry, total energy intake, and the modified total score for each index (total score – component i) in the smoking model.
*Moderation components: higher scores represent lower consumption.
